# Assessment of Risk Factors and Obstetric Outcome of Adolescent Pregnancies Through a Prospective Observational Analysis

**DOI:** 10.7759/cureus.30775

**Published:** 2022-10-27

**Authors:** Anupma Anupma, Avir Sarkar, Neelima Choudhary, Sonam Jindal, Jagadish C Sharma

**Affiliations:** 1 Obstetrics and Gynecology, Employees' State Insurance Corporation (ESIC) Medical College and Hospital, Faridabad, IND

**Keywords:** neonatal outcome, maternal health, high risk obstetrics, teenage pregnancy, adolescent pregnancy

## Abstract

Background

Adolescence is the most crucial stage of life. Early marriage and teenage pregnancy infringe on adolescent girls' social and humanitarian rights. Moreover, it leads to school dropouts and decreased self-autonomy. Through this study, we aimed to analyze the risk factors and obstetric and neonatal outcomes resulting from adolescent pregnancies conceived by Indian girls less than 20 years of age.

Materials and methods

It was a prospective observational study conducted over a period of two years. Consecutive consenting adolescent mothers visiting the antenatal clinic or the delivery wards were recruited into the study. Adolescent pregnancies constituted all pregnancies where the maternal age was between 14 and 19 years at the time of presentation. Participants were followed prospectively till delivery and postpartum visit at six weeks to assess the obstetric and puerperal outcomes. Treating obstetricians asked about the causes responsible for current teenage pregnancy. At the time of delivery, data pertaining to antenatal complications, pregnancy outcome, mode of delivery, and birth weight were noted. All women were counseled for postpartum contraception at the time of delivery. Compliance with postpartum contraception was noted, and reasons for non-acceptance were asked.

Results

A total of 133 antenatal women in the adolescent age group were recruited during the study time frame. The mean age at the time of delivery was 18.4 years. Most of the women were educated between the sixth and 12th standards and belonged to the upper-lower economic class. Early marriage, increased family pressure, and school dropout at a young age were the predominant risk factors for teenage pregnancy in the study population. The majority of them suffered from anemia. Pregnancy-induced hypertension, hypothyroidism, fetal growth restriction, and oligohydramnios were a few other complications seen in adolescent pregnancies. Despite counseling, only 33.8% of adolescent mothers accepted postpartum contraception (any of the standard methods).

Conclusion

Pregnancy has concerning health consequences on adolescent girls and their babies. For example, adolescent mothers face increased risks of pregnancy-induced hypertension, obstructed labor, and puerperal sepsis. So, it is time to create awareness through mass educational campaigns and widespread family planning services.

## Introduction

Adolescence is a crucial stage of life, marking the transition from childhood to adult life [1]. Various national and international health programs have recently brought adolescent issues into the mainstream. According to the fourth National Family Health Survey (NFHS), 7.9% of adolescent women were already pregnant at the time of the survey, with a higher prevalence in rural areas (9.2%). The rate declined to 6.8% in the fifth NFHS. India suffers an economic loss of 7.7 billion dollars annually due to teenage pregnancies, which accounts for 12% of the gross domestic product [2]. If this scenario continues, India is expected to have the biggest national adolescent girl population of 95 million by 2030 [2]. Early marriage infringes on the social and humanitarian rights of these young women, leading to school dropouts, fewer livelihood options, decreased self-autonomy within the family, and domestic violence [3]. Married adolescent girls are less likely to use contraceptives. Poor birth spacing increases the risk of fetal growth restriction and low birth weight infants [4]. Thus, adolescent pregnancy continues to be an important social issue after more than seven decades of independence. Since adolescent pregnancies are a serious threat to the development of a nation, it requires special attention. The focus should be put on assessing the exact incidence and the various risk factors that contribute to adolescent pregnancy in our country. Through this study, we aimed to analyze the various risk factors and obstetric and neonatal outcomes resulting from adolescent pregnancies conceived by Indian girls less than 20 years of age.

## Materials and methods

It was a prospective observational study conducted in an urban tertiary care hospital and teaching institute from Haryana state of Northern India over a period of two years. Informed consent was obtained from all participants. All procedures in the study involving human participants were performed in accordance with the ethical standards of the Institutional Ethics Committee (approval number 134X/11/13/2021-IEC/36) and with the 1964 Helsinki Declaration and its later amendments or comparable ethical standards. Consecutive consenting adolescent mothers visiting either the antenatal clinic or the delivery wards were recruited into the study. Adolescent pregnancies constituted all pregnancies where the maternal age was between 14 and 19 years at the time of presentation. Pregnancies before 14 years of age were preferably excluded due to comparability to other international studies. Inclusion criteria consisted of women less than 20 years of age coming for either routine antenatal visits to outpatient departments or reaching in an emergency to delivery wards. Both married and unmarried girls were proposed to be included in the study. Although participants aged 18 years and above gave written informed consents, those below 18 years were recruited after obtaining written assent. Mothers not willing to give written informed consent or assent, adolescent pregnancies under 14 years of age, and mothers suffering from chronic mental health disorders and medico-surgical disorders likely to affect obstetric outcomes were excluded from the study. All consenting pregnant women recruited into the study were followed prospectively till delivery and postpartum visit at six weeks to assess the obstetric and puerperal outcomes.

Participants were asked about their age at the time of presentation, marital status, age at the time of marriage, parity, education, and economic status. Treating obstetricians asked about the causes responsible for current teenage pregnancy. At the time of delivery, data pertaining to antenatal complications, pregnancy outcome, mode of delivery, birth weight, and "Appearance, Pulse, Grimace, Activity and Respiration" (APGAR) scores of the newborn at one and five minutes after birth were noted. Newborns requiring continuous positive airway pressure, mechanical ventilation, or IV antibiotics were shifted to the neonatal intensive care unit after delivery. All women were counseled for postpartum contraception at the time of delivery through both vaginal and Cesarean routes. Compliance with postpartum contraception was noted, and reasons for its non-acceptance were asked in detail. At the time of routine postpartum follow-up, mothers were asked about their major health concerns during the postpartum period. They were particularly asked about breastfeeding difficulties, and counseling sessions were conducted accordingly. Postpartum depression was screened using the Edinburgh Postnatal Depression Scale, consisting of 10 validated questions with a maximum score of 30. A total score of 13 or higher was considered postnatal depression. All data were tabulated in a Microsoft Excel worksheet (Microsoft, Redmond, WA, USA), and statistical analysis was performed by SPSS version 22.0 (IBM Corp., Armonk, NY, USA). Rates and proportions were calculated for categorical variables, and the same is presented in a tabular format.

## Results

A total of 147 antenatal women in the adolescent age group were potentially eligible for enrollment during the study time frame of two years. Of them, 14 mothers did not give valid written informed consent or assent. So, 133 adolescent mothers were recruited into the study. All participants were married, with a mean age of marriage being 17.5 years (Table 1). The mean age at the time of delivery was 18.4 years. Most of the women were educated between sixth and 12th standards (78; 58.7%) and belonged to the upper-lower socio-economic class (85; 63.9%) according to the modified Kuppuswamy classification. Early marriage (OR 141.24), increased family pressure (OR 9.88), and school dropout at a young age (OR 3.71) were the predominant risk factors for teenage pregnancy in the study population.

**Table 1:** Maternal outcome of adolescent pregnancies during the study time frame.

S. No.	Maternal parameters		Frequency (n=133)
1.	Mean age at the time of delivery		18.4±0.6
2.	Mean age at the time of marriage		17.5±0.4
3.	Marital status	Married	133 (100%)
		Unmarried	0 (0.0%)
4.	Parity	Primigravida	128 (96.2%)
		Multigravida	5 (3.8%)
5.	Education status	Below fifth standard	55 (41.3%)
		Sixth to 12^th^ standard	78 (58.7%)
		Graduation	0 (0.0%)
6.	Socio economic status	Lower	12 (9.0%)
		Upper lower	85 (63.9%)
		Lower middle	34 (25.6%)
		Upper middle	2 (1.5%)
		Upper	0 (0.0%)
7.	Risk factors of adolescent pregnancy	Early marriage	131 (98.4%)
		Family pressure	114 (85.7%)
		School dropout	78 (58.6%)
8.	Total number of antenatal visits	Less than four	108 (81.2%)
		Four and above (at least one in each trimester)	25 (18.8%)
9.	Pregnancy related complications	Pregnancy-induced hypertension	11 (8.3%)
		Gestational diabetes	0 (0.0%)
		Hypothyroidism	13 (9.8%)
		Anemia	69 (51.8%)
		Thrombocytopenia	2 (1.5%)
		Intrahepatic cholestasis of pregnancy	3 (2.2%)
		Fetal growth restriction	8 (6.1%)
		Oligohydramnios	11 (8.3%)
10.	Pregnancy outcome	Abortion	9 (6.8%)
		Preterm delivery	16 (12.0%)
		Term delivery	108 (81.2%)
11.	Still birth		5 (4.1%)
12.	Mode of delivery	Vaginal delivery	90 (72.6%)
		Caesarean section	34 (27.4%)
13.	Postpartum haemorrhage		9 (7.3%)
14.	Difficulty in breastfeeding		89 (74.8%)
15.	Acceptance of postpartum contraception		45 (33.8%)
16.	Reasons for non-acceptance of postpartum contraception	Fear of becoming infertile	37 (42.0%)
		Fear of interference with breastfeeding	34 (38.6%)
		Family pressure	12 (13.6%)
		Not stated any reasons	5 (5.8%)
17.	Postpartum depression		14 (11.8%)

Only 18.8% of adolescent pregnant women visited the antenatal clinic during all three trimesters. The majority of the study population were primigravidae (96.2%). The majority of them suffered from anemia (69; 51.8%). Pregnancy-induced hypertension (8.3%), hypothyroidism (9.8%), fetal growth restriction (6.1%), and oligohydramnios (8.3%) were a few other complications seen in adolescent pregnancies. No cases of congenital fetal malformations were detected sonologically or after delivery. Nine women (6.8%) had first-trimester spontaneous abortions. A high proportion of the study population had preterm delivery (16; 12.0%). The incidence of Cesarean section in the study population was quite high (27.4%), with fetal distress being the most common cause of Caesarean delivery. A total of nine mothers (7.3%) experienced postpartum hemorrhage, which was amenable to medical management and uterine massage. 

Despite counseling for postpartum contraception, only a few women accepted it (33.8%). The main reasons behind the non-acceptance of postpartum contraception (any of the standard methods) were fear of becoming infertile (42.0%), fear of interference with breastfeeding (38.6%), family pressure (13.6%), and reason unstated (5.8%) in decreasing order of frequency. At six weeks postpartum, almost 74.8% of adolescent mothers reported having found difficulty in breastfeeding their babies and so resorted to the addition of top feeds. Fourteen mothers (11.8%) suffered from postnatal depression and were advised counseling in the psychiatry outpatient department.

Neonatal outcome was also noted simultaneously (Table 2). A high proportion of babies born to adolescent mothers were detected to have low birth weight for the respective gestational age (36; 29.1%). While 23.4% of newborns had APGAR scores of less than seven after one minute of birth, with effective resuscitation, only 9.7% of them had a persistent low score even after five minutes of birth. A large number of neonates (38; 28.6%) had to be shifted to the neonatal intensive care unit (NICU) for continuous positive airway pressure, mechanical ventilation, or prolonged IV antibiotics administration. The most common cause of NICU admission was prematurity and its sequelae like respiratory distress syndrome and neonatal sepsis. All newborns successfully survived the NICU stay and returned to their mothers' side after stabilization (the median period of NICU stay being 10.6 days). 

**Table 2:** Neonatal outcome of mothers with adolescent pregnancy. APGAR: Appearance, Pulse, Grimace, Activity and Respiration score.

S. No.	Neonatal outcome		Frequency(n=124)
1.	Weight of newborn	Appropriate for gestational age	88 (70.9%)
		Low birth weight	36 (29.1%)
2.	APGAR scores at one minute	Less than seven	29 (23.4%)
		Seven or more	95 (76.6%)
3.	APGAR scores at five minutes	Less than seven	12 (9.7%)
		Seven or more	112 (90.3%)
4.	Newborns requiring neonatal intensive care unit admissions		38 (28.6%)

## Discussion

Every year, approximately 21 million adolescent girls in developing countries become pregnant, and 12 million give birth, of which 7.7 million are aged below 15 years [5]. The causes of adolescent pregnancy in low-middle income countries (LMIC) are predominantly related to early age of marriage, exposure to domestic or sexual violence, financial constraints, living in communities where early childbearing is a norm, stress and depression, high-risk behavior like smoking and substance abuse, peer pressure to get indulged in intercourse at an early age, difficulty in accessing contraceptives, fear of social stigma, financial constraints, transportation, or restricting laws [5]. Pregnancy becomes a challenge for these teenage mothers. They are less likely to seek prenatal care except in the third trimester [6]. As a result, risks of medical complications, preterm labor, and low birth weight infants are much higher in teenage pregnancies. The risk of obstructed labor is also higher among adolescent mothers due to an underdeveloped pelvis, which, if inadequately supervised in a primary health center in the LMIC with no facilities for emergency Cesarean section, can lead to multiple complications such as genital tract laceration, obstetric fistula, and puerperal sepsis. Adolescent pregnancies also account for 14% of the estimated 20 million unsafe abortions performed yearly, resulting in about 68,000 maternal mortality [7].

Teenage pregnancies are usually associated with high maternal and neonatal mortality and morbidity. It is directly proportional to the adverse economic outcome of a nation [7,8]. Most of the available literature mentions the determinants and outcomes of adolescent pregnancies from the continent of Africa [9-11]. Many communities in India still continue to believe in the early marriage of girls, which is the prime reason for adolescent pregnancy in these women. Though not so uncommon, very few studies have been conducted to assess the obstetric and neonatal outcomes in these high-risk obstetric populations. Parents and family play a pivotal role in early marriage and school dropout, leading to adolescent pregnancies [12]. Causes are varied and differ from urban to rural settings [13,14]. Pregnancy and childbirth-related complications are the leading cause of death among these girls, accounting for 99% of global maternal deaths of women aged 15-49 years [15]. This study has tried to focus on the causes and obstetric outcomes of adolescent pregnancies conceived by Indian girls. It clearly depicted the true scenario in the sub-urban and urban population of Northern India. Due to a lack of awareness and household work, only a handful of women in the study population could come for antenatal visits to the hospitals. Early marriage, family pressure, and school dropout were the predominant risk factors for adolescent pregnancy in our study population. Anemia was the major medical disorder complicating pregnancy. Increased Caesarean delivery and postpartum difficulty in initiation of breastfeeding were some of the significant issues which need medical attention in the adolescent pregnant population. 

Due to a lack of proper guidelines regarding managing adolescent pregnancies during the course of labor, they were managed just like normal adult pregnancies. An intermittent cardiotocograph was used during labor monitoring in an uncomplicated pregnancy. In high-risk cases with medico-surgical comorbidity, continuous fetal health monitoring was done. Both non-pharmacological and pharmacological methods of labor analgesia were administered as these adolescent girls were anticipated to have a low threshold for pain sensation. Non-pharmacological analgesia was administered using encouragement for massage by birth companions. Pharmacological analgesics included IV pethidine, tramadol, morphine, or epidural analgesia.

Just like prior studies in literature, adolescent pregnancies of the index study were also associated with many medical comorbidities [11,13]. Pregnancy-induced hypertension, anemia, fetal growth restriction, and oligohydramnios were the common complications. Women with antenatal anemia (51.8%) were advised to continue oral iron therapy twice daily, and complete blood counts were repeated for two weeks to assess the response to oral iron. Hematology outpatient referral was also ensured for proper evaluation of anemia. However, no cases of puerperal sepsis were detected during postnatal follow-up in the study population. 

The main strength of the study lies in the fact that no prior study from India has tried to assess the obstetric outcomes and contraceptive choices of adolescent mothers over such a large cohort of the population. It creates a gateway to guide the obstetricians to focus more on this high-risk obstetric group. There are a few limitations as well. It was a single-center study. Due to the huge patient load and time constraints, antenatal counseling for postpartum contraception was impossible. This could be one of the major causes of such low acceptance of postpartum contraceptive uptake in the study population.

## Conclusions

Pregnancy has concerning health consequences on adolescent girls and their babies. Adolescent mothers face increased risks of pregnancy-induced hypertension, anemia, labor dystocia, preterm delivery, and difficulty in breastfeeding in the postpartum period. Options for postpartum spacing contraception have to be explained properly to adolescent mothers using an unbiased cafeteria approach. So, it is time to create awareness through mass educational campaigns and widespread family planning services.
